# Myeloperoxidase in Health and Disease

**DOI:** 10.3390/ijms24097725

**Published:** 2023-04-23

**Authors:** Tamara Ortiz-Cerda, Kangzhe Xie, Albaraa Mojadadi, Paul K. Witting

**Affiliations:** 1 Redox Biology Group, Charles Perkins Centre, School of Medical Sciences, Faculty of Medicine and Health, The University of Sydney, Sydney, NSW 2006, Australia; tamara.ortizcerda@sydney.edu.au (T.O.-C.); kangzhe.xie@sydney.edu.au (K.X.); albaraa.mojadadi@sydney.edu.au (A.M.); 2 Department of Normal and Pathological Cytology and Histology, Faculty of Medicine, University of Seville, Avenue Sánchez-Pizjuán s/n, 41009 Seville, Spain

Innate and adaptive immune responses comprise a complex network of protein–protein and protein–cell interactions that regulates commensal flora and protects organisms from foreign pathogens and transformed (proliferating) host cells under physiological conditions such as pregnancy, growth and development as well as formulating a response pathological challenge. Recruitment/activation of inflammatory cells yields enzymatic products that influence innate and adaptive immunity [1,2,3,4]. Neutrophils/monocytes/macrophages express/release the haem enzyme myeloperoxidase (MPO) [5] into the extracellular milieu during NETosis where it principally catalyses hypochlorous acid (HOCl) production. Bactericidal HOCl modulates commensal flora populations and invading pathogens to maintain homeostasis (for example, at sites of intense microbial interaction, e.g., gut mucosal surfaces, Figure 1).

The potent oxidant HOCl also reacts with key amino acids to regulate protein activity/function, such as S100 calgranulin proteins [6]; e.g., HOCl-mediated thiol oxidation in calprotectin alters its capacity to sequester metals and limit bacterial growth [7].

In the Special Issue entitled “Role for the Enzyme Myeloperoxidase to Elicit Pathologies” of the *International Journal of Molecular Sciences* includes a total of seven contributions: five original articles and two reviews have collectively provided new information about the role for MPO in health and disease. For example, Prasch, J. et al. have demonstrated an increase in the formation of lysophospholipid and chlorinated aldehyde such as 2-chlorohexadecanal (2-ClHDA) in the hearts from experimental murine model of endotoxemia. Interestingly, this study revealed that the lipid oxidation/modification resulted from MPO-mediated HOCl production [8]. On the other hand, the work by El Kazzi et al. showed that supplementation with 4 nitroxide 4-methoxyTEMPO (4-MetT), a nitroxide which inhibits HOCl-mediated tyrosine residue modifications, restored the ventricular function by targeting of MPO and TNF-α in experimental acute myocardial infarction in rats [9].

The next paper by Lee J-h et al. investigated the effect of 4,4′-Diaminodiphenyl Sulfone (DDS) as an inflammasome competitor in patients diagnosed with COVID-19. The outcomes indicated that DDS not only reduced the NLRP3 inflammasome pathway, but also blocked MPO and downregulated the production of hypochlorite, which hampered the low pH-dependent steps of viral replication, thereby reducing the inflammatory response [10]. The study by Goeritzer et al. demonstrated that pharmacological inhibition of MPO enzyme using 4-aminobenzoic acid hydrazide (ABAH) or genetic knock out of MPO gene restored disrupted sphingolipid homeostasis and alleviated endothelial cell barrier dysfunction in a lipopolysaccharide-induced murine model of sepsis. The pharmacological inhibition of MPO also lowered circulating level of IL-1β in the septic mice. Interestingly, ABAH inhibition of MPO resulted in higher baseline level of circulating IL-6 and TNF-α in both wildtype and septic mice [11]. The article by San Gabriel P.T. et al. concluded that components with the ability to modulate MPO activity must be carefully considered in the context of each clinical condition. In that regard, thiocyanate (SCN), which has a potential to become a competitive substrate for MPO, favouring the production of the hypothiocyanous acid (HOSCN) oxidant, appears to be protective in respiratory and cardiovascular disease. However, SCN showed pro-arthritic and pro-inflammatory changes in the pathogenesis of rheumatic arthritis, while there was no evidence to support its role in IBD [12].

Critical literature reviews on MPO properties on immune response and pathogenic mechanism have been discussed. The review article from Arnhold J. canvassed the beneficial contributions of MPO in mediating innate immune responses against invading pathogens via phagocytosis and the formation of neutrophil extracellular traps. However, the author also highlighted the potential harmful complications of dysregulated MPO activities in inflammatory conditions such as atherosclerosis, rheumatoid arthritis and inflammatory bowel disease [13].

Finally, the review article by Malecki et al. identified the contributing roles of MPO-associated oxidative stress in disturbing extracellular matrix homeostasis, activating matrix metalloproteinases and ERK1/2 signalling pathways, impairing endothelial cell function and inducing DNA modifications during thoracic aortic aneurysms disease progression [14].

In conclusion, neutrophil-derived MPO is an important factor in host tissue defense, and MPO oxidants play a prominent pro-inflammatory role through promoting host-tissue damage and activating redox-sensitive signalling cascades. Notably, MPO and its oxidants are also important mediators of normal physiological immunity [15,16]. For example, HOCl-modified proteins enhance immunogenicity, and this discovery led to the novel proposal that neutrophils are accessory cells involved in staging adaptive immunity, i.e., neutrophil-MPO provides a link between innate and adaptive responses. In addition to this role in normal physiology, unregulated MPO activity can also result in significant host oxidative tissue damage that can exacerbate pathologies (e.g., refer to Figure 1 above). This duality of action for MPO (host-physiology vs pathophysiological action) has steered contemporary research to identify novel factors that govern MPO activity in different microenvironments, and this has led to the development of specific pharmacological agents that target MPO under varying pathological conditions [17]. Whether targeting MPO for therapeutic gain proves to be useful in inflammation-mediated human disorders remains to be demonstrated by future clinical research.

## Figures and Tables

**Figure 1:**
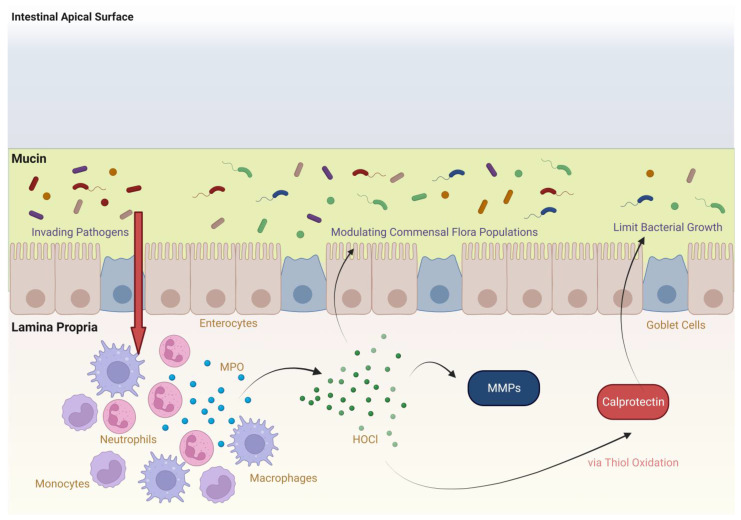
The role of myeloperoxidase in modulating inflammatory responses in gastrointestinal epithelium. This exemplar image illustrates the invasion of pathogens into the colon wall that stimulates the recruitment of immune cells. Release of the haem enzyme MPO from neutrophils, monocytes and macrophages, which in turn catalyses the production of the potent bactericidal agent hypochlorous acid (HOCl). In addition, HOCl interacts with, but is not limited to, matrix metalloproteinases (MMPs) S100 “calgranulin” proteins (such as calprotectin) to modulate commensal floral population, control inflammatory responses and limit bacterial growth. Similar inflammatory responses can be established in the vasculature, joints and other organs in the presence of varying pathogenic stimuli. HOCl: hypochlorous acid; MMPs: matrix metalloproteinases; MPO: myeloperoxidase. Illustration created with www.Biorender.com with appropriate licensing (accessed 20 April 2023).
